# Assessment of PIM-2 performance among surgical patients with heart disease and correlation of results with RACHS-1

**DOI:** 10.5935/0103-507X.20170069

**Published:** 2017

**Authors:** Raíssa Queiroz Rezende, Cláudia Pires Ricachinevsky, Aline Botta, Viviane Rampon Angeli, Aldemir José da Silva Nogueira

**Affiliations:** 1 Hospital da Criança Santo Antônio, Irmandade Santa Casa de Misericórdia de Porto Alegre - Rio Grande do Sul (RS), Brazil.

**Keywords:** Heart defects, congenital/surgery, Heart defects, congenital/mortality, Postoperative period, Risk adjustment, Risk assessment, Cardiopatias congênitas/cirurgia, Cardiopatias congênitas/mortalidade, Período pós-operatório, Risco ajustado, Avaliação de risco

## Abstract

**Objective:**

To assess the performance of the Pediatric Index of Mortality (PIM) 2 and the Risk Adjustment for Congenital Heart Surgery (RACHS) in the postoperative period of congenital heart disease patients.

**Methods:**

Retrospective cross-sectional study. Data were collected from patient records to generate the scores and predictions using recommended techniques, demographic data and outcomes. The Mann-Whitney test, Hosmer-Lemeshow test, standardized mortality rate, area under the receiver operating characteristic (ROC) curve, chi square test, Poisson regression with robust variance and Spearman's test were used for statistical analysis.

**Results:**

A total of 263 patients were evaluated, and 72 died (27.4%). These patients presented significantly higher PIM-2 values than survivors (p < 0.001). In the RACHS-1 classification, mortality was progressively higher according to the complexity of the procedure, with a 3.24-fold increase in the comparison between groups 6 and 2. The area under the ROC curve for PIM-2 was 0.81 (95%CI 0.75 - 0.87), while for RACHS-1, it was 0.70 (95%CI 0.63 - 0.77). The RACHS presented better calibration power in the sample analyzed. A significantly positive correlation was found between the results of both scores (r_s_ = 0.532; p < 0.001).

**Conclusion:**

RACHS presented good calibration power, and RACHS-1 and PIM-2 demonstrated good performance with regard to their discriminating capacities between survivors and non-survivors. Moreover, a positive correlation was found between the results of the two risk scores.

## Abstract (pt)

**Objetivo:**

Avaliar o desempenho do *Pediatric Index of Mortality* (PIM) 2 e do Escore de Risco Ajustado para Cirurgia Cardíaca Congênita (RACHS) no pós-operatório de cardiopatas congênitos.

**Métodos:**

Estudo transversal retrospectivo. Foram coletados dados de prontuário para gerar os escores e predições com as técnicas preconizadas, os dados demográficos e os desfechos. Para estatística, utilizaram-se o teste de Mann-Whitney, o teste de Hosmer-Lemeshow, a taxa de mortalidade padronizada, a área sobre a curva COR, qui quadrado, regressão de Poisson com variância robusta e teste de Spearman.

**Resultados:**

Foram avaliados 263 pacientes, e 72 foram a óbito (27,4%). Estes apresentaram valores de PIM-2 significativamente maiores que os sobreviventes (p < 0,001). Na classificação RACHS-1, a mortalidade foi progressivamente maior, de acordo com a complexidade do procedimento, com aumento de 3,24 vezes na comparação entre os grupos 6 e 2. A área abaixo da curva COR para o PIM-2 foi 0,81 (IC95% 0,75 - 0,87) e, para RACHS-1, de 0,70 (IC95% 0,63 - 0,77). A RACHS apresentou melhor poder de calibração na amostra analisada. Foi encontrada correlação significativamente positiva entre os resultados de ambos os escores (r_s_ = 0,532; p < 0,001).

**Conclusão:**

A RACHS apresentou bom poder de calibração, e RACHS-1 e PIM-2 demonstraram bom desempenho quanto à capacidade de discriminação entre sobreviventes e não sobreviventes. Ainda, foi encontrada correlação positiva entre os resultados dos dois escores de risco.

## INTRODUCTION

Pediatric intensive care units (ICU) provide care for critically ill children through skilled professionals and highly complex therapies. According to the latest census of the *Associação de Terapia Intensiva Brasileira* (AMIB), published in 2010, Brazil has 2,342 ICU, 12.5% of which are pediatric.^(1)^

Evaluating the performance of the care provided in a pediatric ICU is a complex task due to the large number of variables involved in the care of these patients. In addition, several factors not associated with quality of service affect the individual risk of death of these patients, such as the current diagnosis, severity of the acute illness, status of the underlying disease and various additional risk factors.^(2)^ Due to the variability of cases in an ICU, any measure taken globally, such as mortality rate, cannot be interpreted without risk adjustment. To this end, several scoring systems for the quantification of severity and prognosis have been created in recent years.

The main scores for the pediatric population are the Pediatric Index of Mortality (PIM), the Pediatric Risk of Mortality (PRISM) and their new versions. These scores were developed from the identification of variables relevant to mortality risk and upon scoring them after a multivariate statistical analysis using logistic regression.^(3)^

The PIM, originally developed in 1997, is a simple model, based on variables collected at the time of admission to the pediatric ICU. Due to new treatment technologies and new approaches to critical patient care, an updated version of this score (PIM-2) was produced in 2003, resulting from a study that included ICU with large variabilities of diagnoses in Australia, the United Kingdom and New Zealand. The addition of variables that identify diagnoses with low mortality risk improved the performance of the PIM-2 for non-cardiac postoperative patients and respiratory patients.^(4)^

Although the PIM-2 is a widely used mortality score in pediatric ICUs in Brazil and around the world, its applicability is questionable in cases of ICUs with a greater demand for specific diseases, such as congenital heart disease.

Congenital heart disease is an important cause of death in childhood, and the care of these children inspires studies and advances in the area of cardiac surgery and intensive care. According to a systematic review and meta-analysis by van der Linde et al., the prevalence of congenital heart disease is 9.1 per thousand live births.^(5)^ It is estimated that 7% of deaths in the neonatal period are related to congenital heart diseases, corresponding worldwide to approximately 9 million deaths. In addition, it is known that 25% of congenital heart disease patients will require an invasive procedure in the first year of life.^(6)^

Evaluating the quality of the services that perform corrections of congenital heart defects is a difficult task, especially due to the wide variety of existing heart defects. To create a model of risk adjustment for short-term mortality of all types of surgery for congenital heart disease, Jenkins et al. developed a model called the Risk Adjustment Score for Congenital Heart Surgery (RACHS-1), which classifies six categories of risk that allow the comparison of in-hospital mortality for groups of children undergoing surgery for congenital heart disease.^(7)^

The objective of this study was to test the validity of the PIM-2 in the subpopulation of postoperative congenital heart disease patients in our ICU and to compare its results with a specific score developed for this population (RACHS-1).

## METHODS

This retrospective cross-sectional study analyzed the medical records of eligible cases from 2015. Patients undergoing cardiac surgery admitted to the pediatric ICU of the *Hospital da Criança Santo Antônio*, located in Porto Alegre, Rio Grande do Sul (RS), Brazil, were considered eligible. Cases of intraoperative death due to the impossibility of calculating the PIM-2, postoperative recovery in another unit or cardiac surgeries not classified by the RACHS-1 were excluded from the study. Data were collected retrospectively to generate the scores and predictions with the recommended technique (for the PIM-2, data from the first hour of hospitalization; for the RACHS-1, identification of the underlying heart disease and corrective surgery performed). In addition, demographic data were collected to characterize the sample, including age at admission, gender, weight, length of hospital stay, diagnosis of Down syndrome and use of extracorporeal circulation. The outcome evaluated was patient evolution (death, hospital discharge or surgical reintervention).

Simple descriptive analysis was used to characterize age, weight and length of hospital stay. The other variables were described using absolute frequency and percentage. The correlation between the patients who died and the results found in the PIM-2 was evaluated using the Mann-Whitney test. For comparison of mortality between the RACHS-1 groups, the chi-square test was used, and the prevalence ratios were obtained from the Poisson regression analysis, with robust variance. The calibration of the PIM-2 and RACHS-1 by death probability ranges was assessed using the Hosmer-Lemeshow test, the logistic regression model and the standardized mortality rate, with a 95% confidence interval (95%CI) for each risk range. The capacity to discriminate between survivors and non-survivors was assessed using the receiver operating characteristic (ROC) curve. The associations between the results of the PIM-2 and RACHS-1 were tested using the chi-square test, and the quantitative correlation between the scores was analyzed using the Spearman test. Data analysis was performed with the Statistical Package for the Social Sciences (SPSS) version 23.0.

The project was approved by the Ethics and Research Committee of the *Hospital da Criança Santo Antônio* - *Irmandade Santa Casa de Misericórdia de Porto Alegre* (RS) (Opinion 1,745,596, CAAE 58957516.6.0000.5683).

## RESULTS

**Figure 1:**
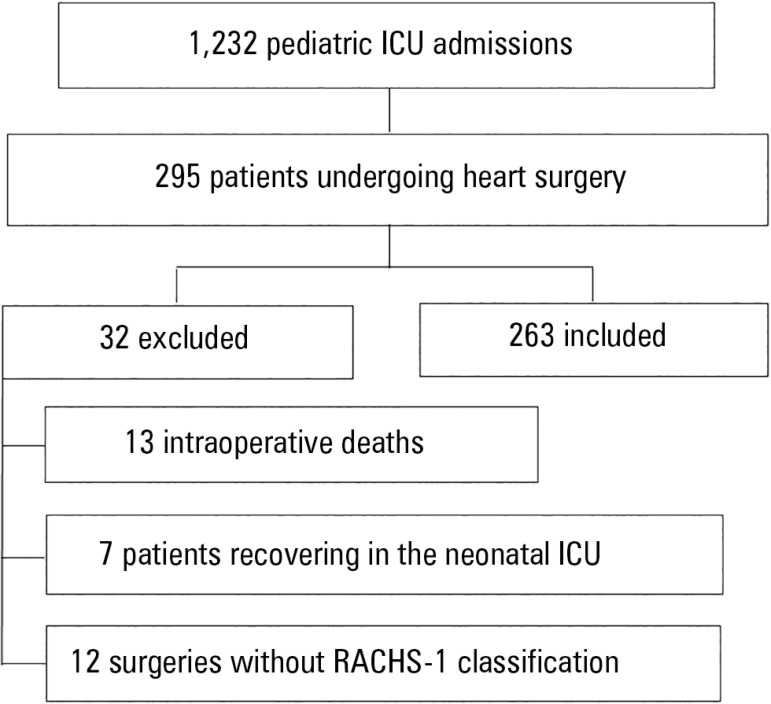
Inclusion and exclusion criteria for the sample. ICU - intensive care unit; RACHS - Risk Adjustment for Congenital Heart Surgery.

Patients admitted to the pediatric ICU of the *Hospital da Criança Santo Antônio* from January to December 2015 were evaluated. The service had 30 beds and nursed critically ill children with various pathologies and congenital heart disease patients. During 2015, 1,232 hospitalizations were performed. The medical records of 295 patients undergoing heart surgery were analyzed; of these, 32 were excluded due to intraoperative death, recovery in the neonatal ICU or heart surgeries without RACHS-1 classification, such as cardiac pacemaker implantation (Figure 1).

A total of 263 congenital heart disease patients were eligible for the study in the immediate postoperative period of heart surgery. When analyzing the profile of the sample, 62.4% of the patients were male, the median age was 5 months, 6.5% were diagnosed with Down syndrome, and the mean length of hospital stay was 11.4 days. Regarding patient origin, 35.4% were from the interior of the state or were transferred from other Brazilian states. Among the surgeries evaluated, 76% used extracorporeal circulation. Of the 263 patients studied, 72 died (27.4%) (Table 1).

**Table 1:** Sample characteristics (n = 263)

Profile	N (%)	Median	25^th^ - 75^th^ Percentiles
Male gender	164 (62.4)		
Age (months)		5	0 - 24
< 30 days	77 (29.3)		
≥ 30 days	186 (70.7)		
Weight (kg)		5	3.2 - 10
Length of stay in the pediatric ICU (days)		6	2 - 14
Origin			
Porto Alegre	170 (64.6)		
Other cities/states	93 (35.4)		
Down syndrome	17 (6.4)		
With ECC	200 (76)		
Outcome			
Death	72 (27.4)		
Discharge from pediatric ICU	178 (67.6)		
Surgical reintervention	13 (4.9)		

ICU - intensive care unit; ECC - extracorporeal circulation.

**Figure 2:**
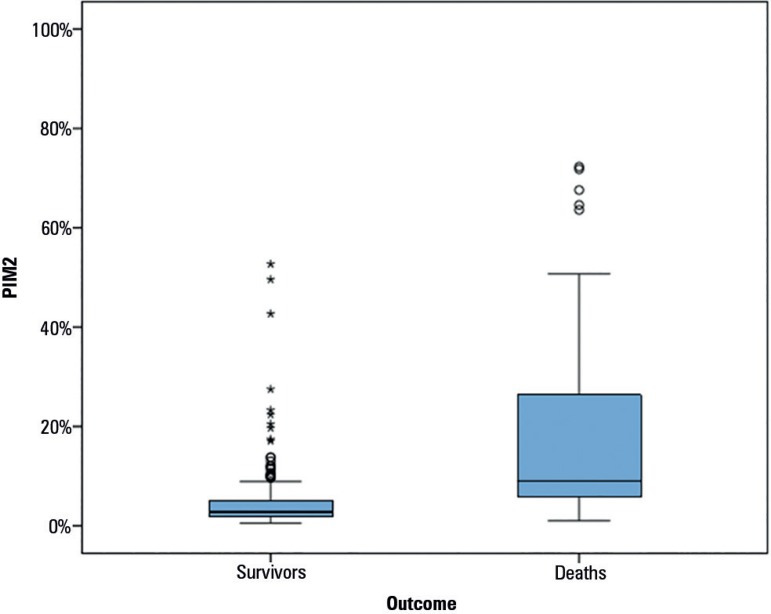
Mann-Whitney test for assessment of the Pediatric Index of Mortality 2 according to outcome. PIM - Pediatric Index of Mortality.

When separated by outcome, the PIM-2 scores of the patients who survived ranged from 0.5 to 52.7%, with a median of 2.8%, whereas the PIM-2 scores of the patients who died ranged from 1 to 72.3%, with a median of 9.1%. Using the Mann-Whitney test, it was determined that patients who died had a significantly higher PIM-2 score than patients who survived (p < 0.001) (Figure 2).

When classifying the cases into RACHS-1 risk categories, the patients were distributed as described in table 3. For comparison of mortality among the groups of the RACHS-1 scale, the chi-square test was used, and prevalence ratios were obtained using Poisson regression with robust variance. Using group 2 as a reference (as there were no deaths in group 1), groups 3, 4 and 6 showed increases in mortality of 54.7% (p = 0.109), 95.8% (p = 0.031) and 224% (p < 0.01), respectively (Table 2).

**Table 3:** Calibration of the Pediatric Index of Mortality 2 and the Risk Adjustment for Congenital Heart Surgery

	Number of patients	Observed survival	Expected survival	Observed deaths	Expected deaths	SMR (95% CI)
PIM-2						
0 - 1	16	15	13.58	1	2.42	0.41 (0.01 - 2.30)
> 1 - 5	143	128	118.26	15	24.74	0.61 (0.34 - 1.00)
> 5 - 15	71	38	52.4	33	18.60	1.77 (1.22 - 2.49)
> 15 - 30	15	7	6.33	8	8.67	0.92 (0.40 - 1.81)
> 30	18	3	1.17	15	16.83	0.89 (0.50 - 1.47)
Hosmer-Lemeshow goodness-of-fit test				χ^2^ = 6.13; p = 0.047		
RACHS-1						
1	27	27	24.15	0	2.85	0.0 (0.0 - 1.29)
2	94	76	77.90	18	16.10	1.12 (0.66 - 1.77)
3	81	57	59.49	24	21.51	1.12 (0.72 - 1.66)
4	32	20	19.60	12	12.40	0.97 (0.50 - 1.69)
6	29	11	9.87	18	19.13	0.94 (0.56 - 1.49)
Hosmer-Lemeshow goodness-of-fit test				χ^2^ = 4.07; p = 0.254		

PIM - Pediatric Index of Mortality; RACHS-1 - Risk Adjustment for Congenital Heart Surgery; SMR - standardized mortality rate; 95%CI - 95% confidence interval.

**Table 2:** Comparison of mortality rates found among groups according to the Risk Adjustment for Congenital Heart Surgery

RACHS-1	Rate N (%)	Deaths N (%)	PR	95%CI	p value	Mortality expected by the RACHS-1 (%)
1	27 (10.2)	0 (0)	-	-	-	0.4
2	94 (35.7)	18 (19.1)	1	-	-	3.8
3	81 (30.7)	24 (29.6)	1.54	0.90 - 2.63	0.109	8.5
4	32 (12.1)	12 (37.5)	1.95	1.06 - 3.60	0.031	19.4
5	-	-	-	-	-	-
6	29 (11.0)	18 (62.1)	3.24	1.95 - 5.36	<0.001	47.7
Total	263 (100)	72 (27.4)	-	-	-	-

RACHS - Risk Adjustment for Congenital Heart Surgery; PR - prevalence ratio; 95%CI - 95% confidence interval.

**Figure 3:**
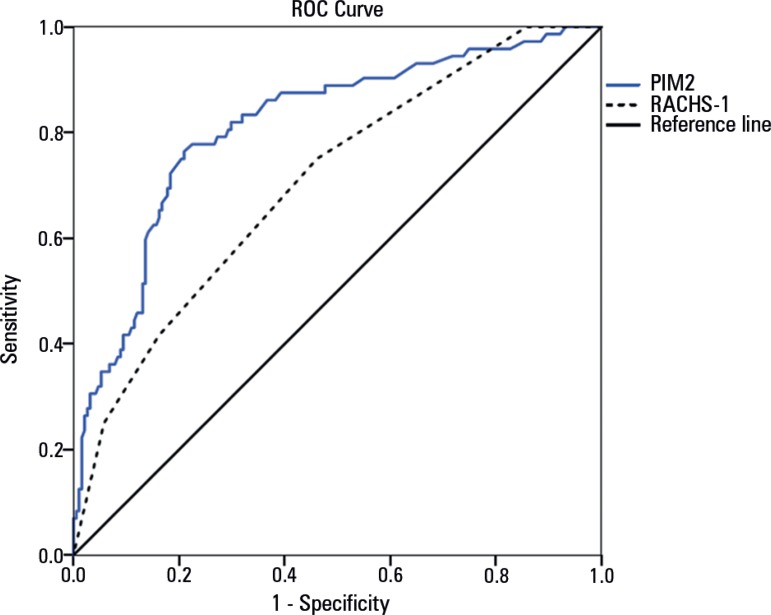
Assessment of the discriminatory performance of the models according to the area under the receiver operating characteristic curve. ROC - receiver operating characteristic; PIM - Pediatric Index of Mortality; RACHS - Risk Adjustment for Congenital Heart Surgery.

**Figure 4:**
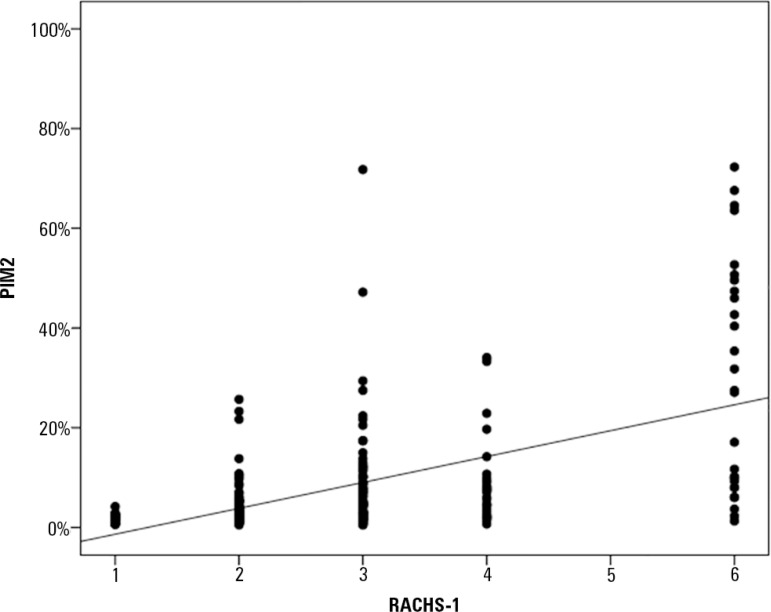
Correlation between the Pediatric Index of Mortality 2 and the Risk Adjustment for Congenital Heart Surgery 1, according to the Spearman test. PIM - Pediatric Index of Mortality; RACHS - Risk Adjustment for Congenital Heart Surgery.

The scores were calibrated using the Hosmer-Lemeshow goodness-of-fit test, which showed significance for PIM-2, with a chi-square of 6.13 (p = 0.047), and non-significant values for RACHS-1, with a chi-square of 4.07 (p = 0.254). The standardized mortality rates were also calculated per death probability ranges and are described in table 3. In the discriminatory performance of the models, measured by the area under the ROC curve, areas of 0.81 for PIM-2 (95%CI 0.75 - 0.87) and 0.70 for RACHS-1 (95%CI 0.63 - 0.77) were obtained (Figure 3). The Spearman test was performed to quantify the correlation between the results of the PIM-2 and RACHS-1 scores, and a significant positive correlation was found (r_s_ = 0.532, p < 0.001) (Figure 4).

## DISCUSSION

The mortality rate of 27.4% in the analyzed sample was higher than that found in the literature. In a study on the performance of pediatric heart surgery in the state of São Paulo, a mean hospital mortality rate of 14% was described, with 26.8% among neonates and 9.32% among children from 29 days to 1 year of age.^(8)^ Factors related to the studied sample may have led to increased mortality, such as the considerable proportion of neonates (29.3%), along with factors not evaluated in this study, such as comorbidities, malnutrition and duration of extracorporeal circulation.^(9)^ In addition to intrinsic factors, it is important to assess external factors that affect the quality of heart surgery services in Brazil, with emphasis on delayed diagnosis of these patients, difficulty accessing specialized centers and lack of investment and capacity building.^(8)^ In our service, approximately one-third of the patients came from the interior of the state or from other states, which may result in delayed diagnosis and consequent clinical deterioration. It is also worth noting that in 2015, our hospital did not yet have the capacity to routinely establish extracorporeal membrane oxygenation, a factor that could lead to higher postoperative survival.

The PIM-2 is a score validated in pediatric ICUs worldwide. In Brazil and Latin America, some services tested the performance of the PIM-2 and obtained varied results, but in general, the PIM-2 had good discriminatory capacity and regular calibration.^(10-13)^ However, when the population of a unit tends to present a particular disease, the predictive capacity of a risk model may become less specific.^(14)^

In our population of immediate postoperative congenital heart disease patients, when assessing the calibration power using the Hosmer-Lemeshow test, the differences between the observed mortalities and mortalities expected by the PIM-2 were significant (p = 0.047), demonstrating poor calibration for the PIM-2. This finding was corroborated by the mortality ratio, which presented better prediction only for PIM-2 scores above 15. The discriminatory performance assessed using the ROC curve analysis demonstrated that the PIM-2 showed good discrimination capacity between survivors and non-survivors, with an area under the ROC curve of 0.81 (95%CI 0.75 - 0.87). Few studies have evaluated the performance of the PIM-2 for surgical congenital heart disease patients. Czaja et al. were the pioneers in this effort and retrospectively analyzed a multicenter pediatric ICU database in the United States from 2005 to 2007 and found that, despite the good discriminatory capacity, the PIM-2 underestimated mortality for preoperative cardiac cases while also overestimating the expected number of deaths for the perioperative cardiac group (patients operated on < 24 hours after admission or immediate postoperative patients).^(14)^ An Italian study evaluated ICU admissions from 2009 to 2011 and, when separately analyzing the heart surgery group, found that the number of deaths expected from the application of the PIM-2 for high-risk groups was four times higher than that observed. The consistency of the results found in these two studies suggested that the calibration of the PIM-2 for cardiac surgical patients may be suboptimal.^(15)^ A similar result was found by Jones et al. in a population of postoperative corrective cardiac surgery patients in the United Kingdom, which demonstrated good discriminatory power but poor calibration for the PIM-2.^(16)^

Unlike the PIM-2, the RACHS-1 was created to predict the mortality of a specific group of patients: surgical congenital heart disease patients. When classifying our patients according to the RACHS-1 scale, progressively higher mortality was found as the complexity of the procedure on the scale increased. There were no deaths in group 1, which made it impossible to compare the other groups to this group. Therefore, in comparison to group 2 (used as a reference), the later groups presented progressively higher mortality, with group 6 presenting a 3.24-fold higher mortality rate than group 2. No patients were classified into group 5, as the two pathologies are rare (correction of Ebstein anomaly at age ≤ 30 d and repair of truncus arteriosus and interrupted aortic arch).

It is important to note that the analysis of the sample in relation to the RACHS-1 was performed to observe the increase in mortality according to surgical complexity and severity of heart disease, according to the classification proposed by Jenkins et al. We did not intend to correlate the numbers found in our sample with the original model, as the original was created based on data from the Pediatric Cardiac Care Consortium, which brings together data from American reference centers, which have much more extensive medical resources and experience in cardiac surgery than that found in our environment, which, consequently, resulted in mortality rates much lower than those found in our study and in other Brazilian services.^(7)^

The RACHS-1 has been widely applied in several countries with positive correlations, including in Brazil. In the present study, the RACHS-1 presented good calibration power according to the Hosmer-Lemeshow test, as the differences between the observed and expected values were not significant (p = 0.254). This finding was corroborated by the standardized mortality ratio, in which values closer to 1 were obtained by the RACHS-1, except in the first category. Moreover, the RACHS-1 presented good discriminatory power between deaths and survivors, with an area under the ROC curve of 0.70 (95%CI 0.63 - 0.77). Recently, a Brazilian study applied the RACHS scale to 3,201 patients and found good discriminatory power for hospital mortality, with an area under the ROC curve of 0.754.^(17)^ In a study to validate the RACHS-1 scale in a hospital in London, Kang et al. identified that age at time of surgery, RACHS-1 category and duration of extracorporeal circulation were important risk factors for mortality in children undergoing open heart surgery.^(18)^ A Danish study applied the RACHS to the population of a hospital and found good correlation for in-hospital mortality and length of stay in the ICU.^(19)^

When comparing the performances of the two evaluated scales, we found a significantly positive correlation between the results. No previous studies were found comparing PIM-2 and RACHS-1 scores. A Finnish study, which retrospectively evaluated 1,001 patients after heart surgery to correct congenital heart disease, compared the performance of the RACHS-1 with PRISM, and both scores overestimated the mortality rate.^(20)^

## CONCLUSION

The PIM-2 and RACHS-1 measures demonstrated good performance regarding the capacity to discriminate between survivors and non-survivors in a population of postoperative congenital heart disease patients in a pediatric service in southern Brazil. The PIM-2 presented a greater discriminatory power when the area under the ROC curve was evaluated, whereas the RACHS-1 presented better calibration in the sample studied. Furthermore, a significantly positive correlation was found between the results of the two risk scores.
